# Identification of a KRT9 gene variant and preimplantation genetic diagnosis in a Chinese family with KRT9-palmoplantar epidermal differentiation disorder

**DOI:** 10.1186/s41065-025-00552-y

**Published:** 2025-09-24

**Authors:** Dan Lin, Na Lin, Yao Zhou, Qi Li, Yanlin Ma

**Affiliations:** Key Laboratory of Reproductive Health Diseases Research and Translation of Ministry of Education & Key Laboratory of Human Reproductive Medicine and Genetic Research of Hainan Province & Hainan Provincial Clinical Research Center for Thalassemia, The First Affiliated Hospital of Hainan Medical University, Hainan Medical University, Haikou, 571101 Hainan China

**Keywords:** *KRT9*, Missense mutation, Whole-exome sequencing, PGT-M, Prenatal diagnosis

## Abstract

**Objective:**

To investigate a Chinese family with epidermolysis bullosa palmoplantar keratosis, analyze the mutation loci in this family lineage, and perform preimplantation genetic testing using assisted reproductive technology to enable affected members of this Chinese family to have unaffected offspring.

**Methods:**

Clinical information and blood samples were collected from all affected family members to extract genomic DNA. We detected a mutation site in the KRT9 gene through whole-exome sequencing, then verified this family line’s Keratin-9 gene variant locus using Sanger sequencing. After the pathogenicity was clarified, blastocyst trophoblast cells were extracted for Preimplantation Genetic Testing for Monogenic (PGT-M)(Single Gene) Disorders using the in vitro fertilization embryo transfer technique, and suitable embryos were selected for transfer. Amniocentesis was performed to extract fetal exfoliated cells for prenatal diagnosis at 18 weeks of fetal development.

**Results:**

A heterozygous mutation c.503T > C (p. Leu168Ser), which results in the substitution of a leucine for a serine (p. Leu168Ser), was detected in the *KRT9* gene in the proband and his father, which is located in the highly conserved helix 1 A region of Keratin 9, resulting in an abnormal function of the intermediate filamentous proteins expressed by Keratin 9 encodes genes which are expressed in the palmo-plantar regions of the epidermis, and the patients of the family present with pronounced palmar-plantar keratoderma.

**Conclusion:**

We identified the c.503T > C (p. Leu168Ser) missense mutation in exon 1 of the *KRT9* gene as the cause of *KRT9*-palmoplantar epidermal differentiation disorder (*KRT9*-pEDD) in a Chinese family. Under the guidance of comprehensive genetic counseling, employing PGT-M, we successfully prevented the transmission of the *KRT9*-pEDD pathogenic variant, resulting in the birth of a healthy child.

## Background

*KRT9*-pEDD is an autosomal dominant disorder characterized by diffuse thickening of the skin on the palms and soles [1]. The worldwide incidence of *KRT9*-pEDD is estimated at 2.2 to 4.4 cases per 100,000 live births [2]– [3]. *KRT9*-pEDD is primarily caused by mutations in the Keratin 9 (*KRT9*) gene, with a few instances attributed to mutations in KRT1 [4]. More than 30 mutations in the *KRT9* gene have been identified in *KRT9*-pEDD [5]. *KRT9* is a type I intermediate filament exhibiting tissue-specific expression, predominantly localized to the supranasal keratinocytes of palmoplantar epidermis [6]. *KRT9* mutations are concentrated in the 1 A and 2B helix regions, hot spots for pathogenic changes [7]. Mutations in the 1 A region, in particular, disrupt keratin network formation, leading to severe clinical manifestations [8].

*KRT9*-pEDD typically manifests within the first few weeks to months after birth and persists throughout life. It is often accompanied by a lifelong decrease in sensitivity to heat, tactile sensitivity, difficulty walking, and social challenges. Currently, there is no effective cure for this condition [9]. Prenatal molecular diagnosis or preimplantation genetic testing for monogenic diseases (PGT-M) is crucial for affected individuals to have healthy offspring. This study analyzed a clinically collected Chinese pedigree with *KRT9*-pEDD and identified an additional confirmed case carrying a known pathogenic variant in the *KRT9* gene. This finding expands our understanding of *KRT9*-pEDD and, through genetic counseling, helps reduce the risk of disease recurrence in future offspring of this family.

## Materials and methods

### Patients

**Fig. 1:**
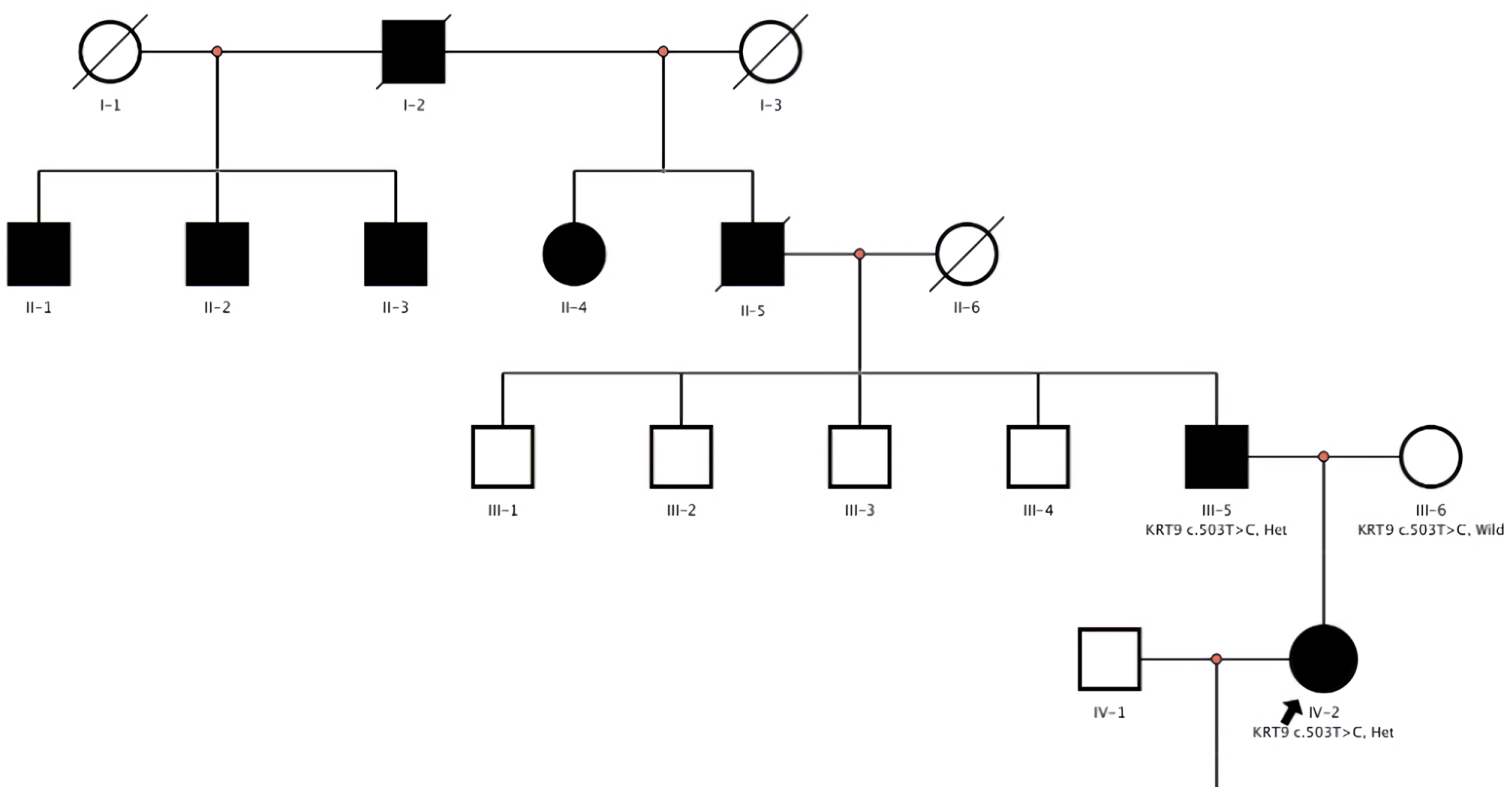
Pedigree analysis of this Chinese family.

In this *KRT9*-pEDD family line, consisting of seventeen relatives across four generations, there were eight patients with *KRT9*-pEDD, including six males and two females (Fig. 1). All patients in the family line experienced disease onset within one year of birth, worsening the condition with age. It manifested as diffuse hyperkeratosis of the entire epidermis of the palms, presenting with a yellowish discoloration and localized scaling and chapping at the lesion sites. The proband was diagnosed with *KRT9*-pEDD at birth, and was married in 2020 with a normal.

**Fig. 2:**
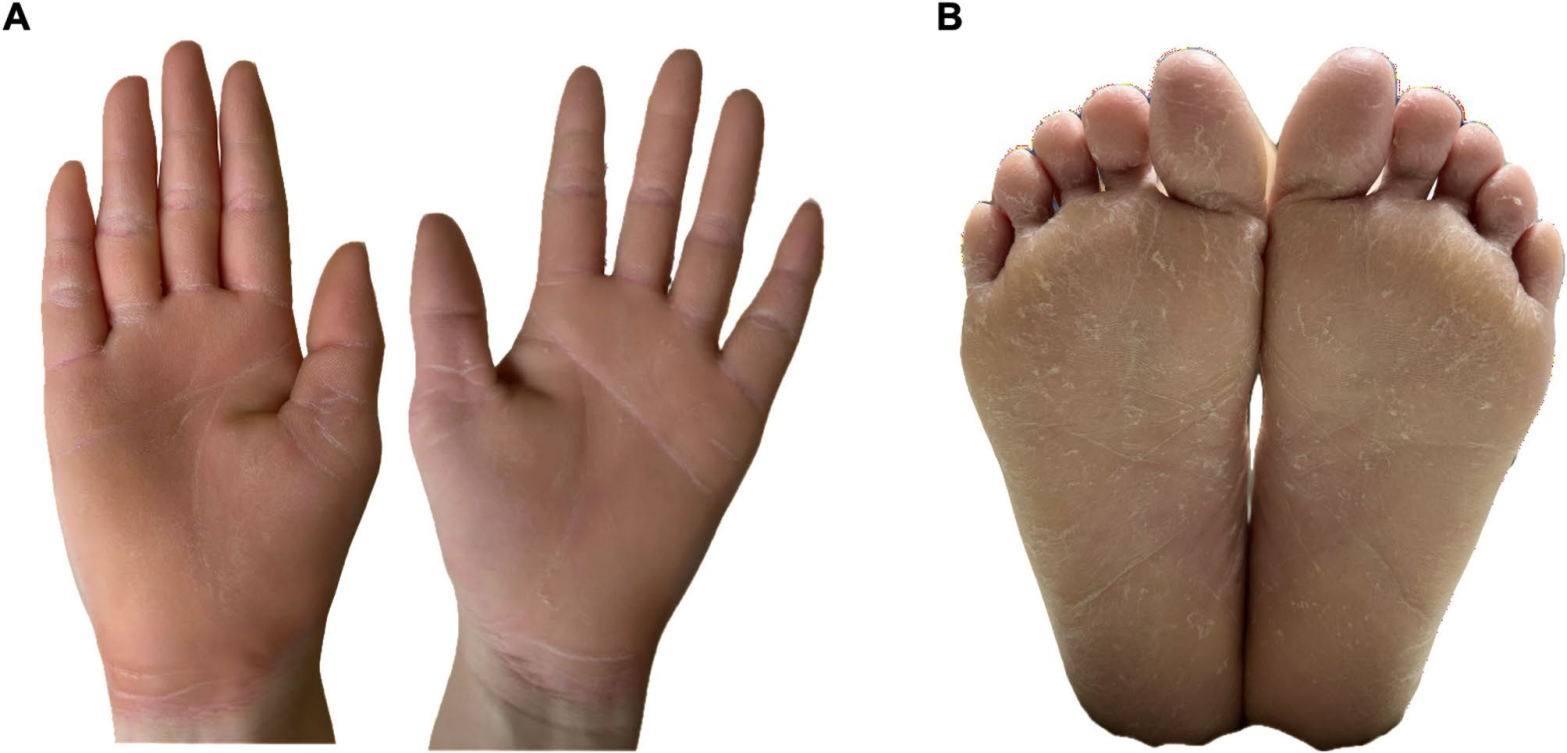
The features of the proband with *KRT9* -pEDD. The diffuse, yellowish thickening of the palmoplantar skin was observed, with well-defined erythematous borders surrounding the affected areas.

**Fig. 3:**
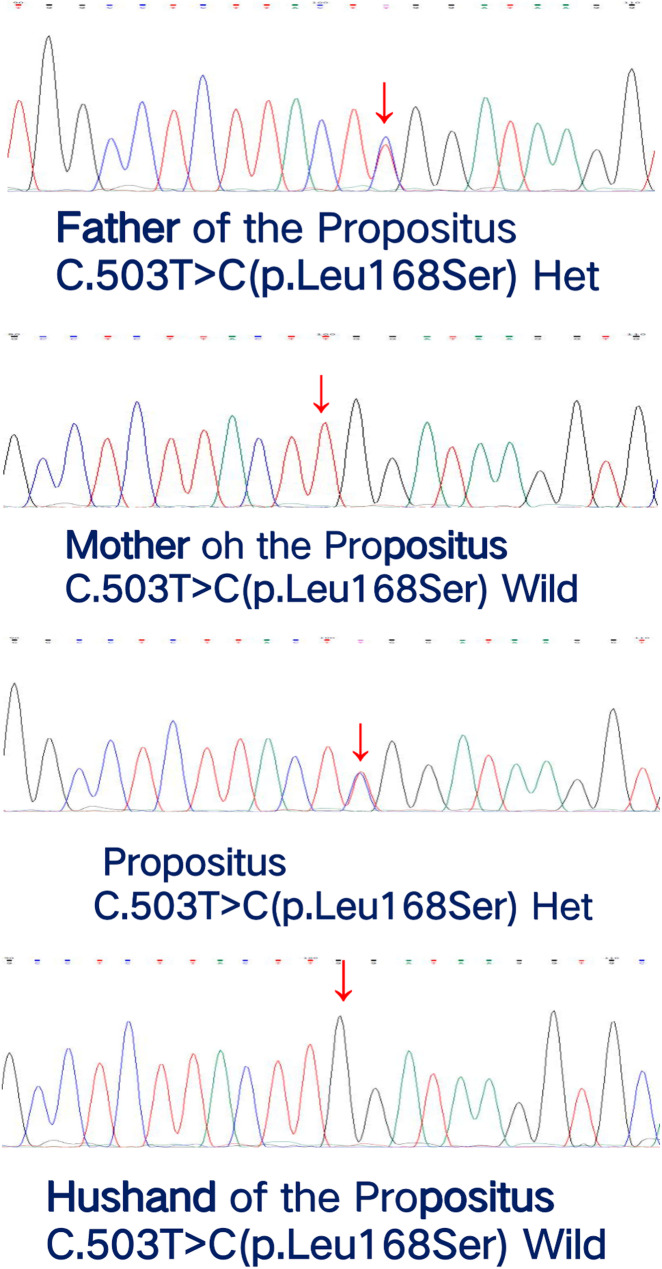
Sanger sequencing revealed that the variation occurred in the *KRT9* gene.

**Fig. 4:**
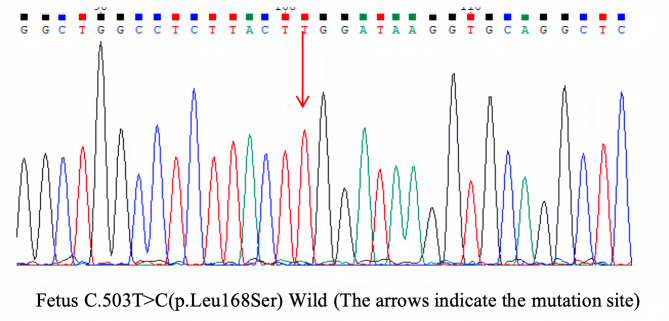
The results of prenatal diagnosis. Sanger sequencing revealed that the fetus did not carry the *KRT9* c.503T > C mutation.

**Fig. 5:**
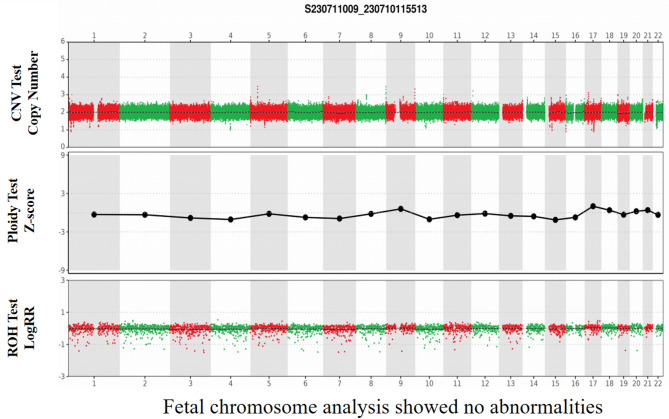
The results of prenatal diagnosis. Genome-wide copy number variation (CNV) analysis was normal.

**Fig. 6:**
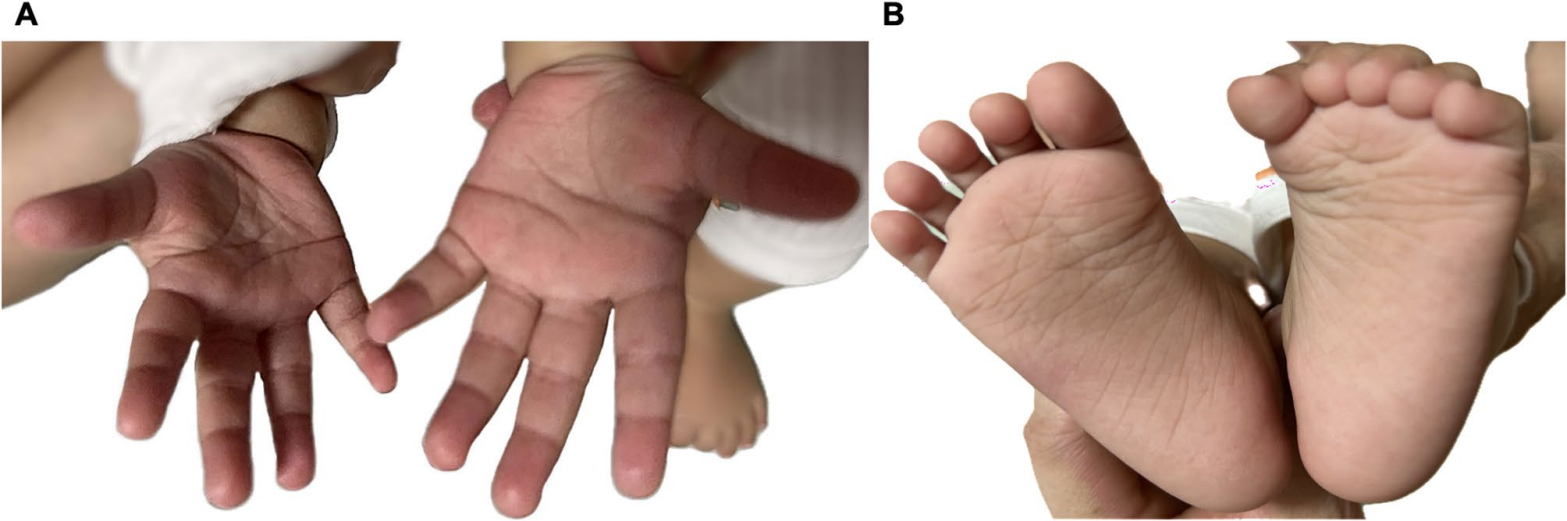
The infant exhibited no clinical signs of *KRT9*-pEDD.

Sex life and has been using contraception since marriage. In 2021, the proband visited our department for genetic counseling to give birth to a healthy child without *KRT9*-pEDD disease.

### Ethics statement

This study was approved by the institutional ethics committee of The First Affiliated Hospital of Hainan Medical University (Ethical approval number: 2023-KYL-264). Informed consent was obtained from the proband and their family for using clinical information and sample collection in this study. Peripheral blood samples and blastocyst trophoblast cells were collected from each study subject. All research procedures adhered to international ethical standards established by the Declaration of Helsinki (1964) and its subsequent amendments while complying with relevant regulations from China’s National Health Commission regarding human genetic resources and assisted reproductive technologies.

### Sample collection and variant validation

We collected 4 mL of peripheral blood samples from the patient, her spouse, and her parents using EDTA anticoagulant tubes (Shenzhen MEDSOON Medical Laboratory Technology Co., Ltd.). Genomic DNA was extracted from the patient’s leukocytes using the QIAGEN kit (Qiagen, Germany) following the manufacturer’s protocol. WES was conducted by a commercial next-generation sequencing (NGS) and molecular diagnostics laboratory. The exome-captured libraries were sequenced using the MGISEQ-2000 platform (BGI, Shenzhen, China) with paired-end sequencing according to the manufacturer’s instructions. Then, the genomic DNA of the patient, her spouse, and her parents was amplified by conventional PCR, purified by commercially available kits, and subjected to Sanger sequencing in both directions to validate and detect variants in the *KRT9* gene. We used Primer 3.0 software to design the *KRT9* gene (NM_000226.3) to identify the target gene (chr17:39722088–39728311 reverse transcription) and upstream and downstream specific SNP sites in the family line. Primers were synthesized by Shanghai Sage Technology (CHINA)Co. Sequences of the primers are: *KRT9*c.503-F: GTGGCTATGGGAGTGGGTTT, *KRT9*c.503-R: GGGGAGTAGTTCTTCTGGATAGC PCR amplification products were sequenced with an ABI3500 sequencer (Applied Biosystems, USA), and Seqman software Chromas 2.6.6 (Technelysium, South Brisbane, QLD, Australia) was used to view the Sanger sequencing results. And compared with the sequence of NG_008300.2 from the NCBI gene bank (USA) to detect mutations in the *KRT9* gene c.503T > C (p. Leu168Ser), reference genome (GRCh37.hgl9).

### PGT-M and prenatal diagnosis

The causative gene was identified in this family, and the pedigree was sufficiently complete to allow haplotype construction. The proband was advised to undergo PGT-M for assisted conception. Ovulation was promoted using gonadotropin-releasing hormone (GnRH) agonists, mature follicles were monitored by ultrasound, oocytes were extracted by puncture, and finally, all stage II oocytes were fertilized by intracytoplasmic monosperm microinjection (ICSI). On day 5 after fertilization, all embryos were fully expanded and cultured to the blastocyst stage, which required blastocyst trophoblast ectoderm (TE) cell biopsy. An annular opening was made in the zona pellucida, and 5 TE cells were aspirated with a biopsy pipette, obtaining 5 TE cells per embryo. The extracted specimens were then lysed, and the biopsied TE cell clusters were rinsed with PBS and collected with 5 µL of lysis buffer into 0.2 mL PCR tubes. Blastocysts were quickly vitrified and frozen using the Kitazato Vitrification Freezing Method after TE cell extraction. The whole-genome amplification of TE cells was performed using the Pico PLEX WGA Kit (New England Biolabs-WGA, US) according to the manufacturer’s protocol. Sequencing libraries were constructed using the Personalized Genome Library Construction Kit (zykw-c-003) from Beijing Jerehao Pharmaceutical Technology Co. Sequencing was performed using Illumina microarrays with a detection resolution of 4 Mb.

Prenatal diagnosis was performed via amniocentesis after obtaining informed written consent at 18 weeks of gestational age and above. Fetal DNA was extracted from 20 mL of amniotic fluid, which was collected under ultrasound guidance for genome-wide CNV testing using the QIAamp DNA Mini Kit (Qiagen, Germany). Bidirectional sequencing was performed using the Sanger sequencing method, and the sequencing results were analyzed according to previously established procedures.

## Results

The hyperkeratosis was inherited in an autosomal-dominant pattern in the pedigree (Fig. 1). All affected individuals exhibited comparable symptomatology. Epidermal hyperkeratosis began at approximately one year of age. The diffuse, yellowish thickening of the palmoplantar skin was observed, with well-defined erythematous borders surrounding the affected areas (Fig. 2). We subjected them to Sanger sequencing in both directions to validate and detect variants in the *KRT9* gene. The results are presented in Fig. 3.

Through the follicular phase long protocol, down-regulated ultra-hyperextension, 33 eggs were retrieved, and four blastocysts were raised (Table 1). By using Next-Generation Sequencing (NGS) and Sanger sequencing, the embryo testing results showed that embryo number 16 was aneuploid but carried the *KRT9* c.503T > C heterozygous mutation, and Embryo number 15 did not bring the *KRT9* c.503T > C mutation but had an abnormal chimeric embryo. And the proband requested the transfer of Embryo number 15. The patient thawed and transferred Embryo number 15 (5-4AA) on March 21, 2023.

**Table 1:** The results of embryo testing by Next-Generation Sequencing (NGS) and Sanger sequencing.

Embryo number	Embryo grading	PGT-A	KRT9c.503T > C (*p*.Leu168Ser)	Portable or not
14	5−4AA	-(mosaic)(6)(q22.31−927)(50.72 Mb)(38%)-(mosaic)21(52%)	—	**—**
15	5−4AA	+(mosaic)(11)(q12.3-q25)(72.45 Mb)(36%)	Wild	**May**
16	5−4AB	Balance	Het	**No**
23	6−4CB	-(mosaic)3(32%);-(mosaic)4(38%)-(mosaic)8(34%);+(mosaic)13(46%)−15;-(mosaic)16(31%)	—	**—**

The couple was closely monitored during the subsequent embryo thawing and transfer cycle to follow their pregnancy status. For intrauterine pregnancies undergoing PGT-M, amniocentesis is recommended for prenatal diagnosis of fetal decidualized cells extracted for genetic testing [10]. Prenatal diagnosis was performed at 18 weeks of gestational age and above, and fetal DNA was obtained from amniotic fluid for genome-wide copy number variation (CNV) analysis and Sanger sequencing. (Figures 4 and 5). An ultrasound performed at our hospital during labor and delivery on April 15, 2023, indicated a singleton intrauterine pregnancy with visible germ and heart tube pulsations. On recent follow-up, the patient delivered a girl by cesarean section on February 8, 2024, at 39 weeks and 6 days of gestation. The infant, now 18 months old, exhibited no clinical signs of *KRT9*-pEDD during two recent follow-up examinations. (Fig. 6).

## Discussion

The proband and family members exhibited typical diffuse palmoplantar hyperkeratosis in our study. Further pedigree analysis revealed that the condition was caused by the c.503T > C (p. Leu168Ser) missense mutation in the *KRT9* gene, leading to *KRT9*-pEDD. This variant is an extremely low-frequency missense mutation in the population, which has been previously reported in the professional version of the Human Gene Mutation Database (HGMD). Based on the pedigree analysis, the disease follows an autosomal dominant inheritance pattern, consistent with this disorder’s known genetic characteristics. In this family, affected individuals presented with typical symptoms weeks to months after birth. After identifying the causative mutation of *KRT9* c.503T > C (p. Leu168Ser), PGT-M enabled selection and transfer of mutation-free embryos, successfully preventing disease transmission. Prenatal and postnatal analyses confirmed the absence of the mutation and associated phenotypes.

The c.503T > C (p. Leu168Ser) variant is located in exon 1 of the KRT9 gene on chromosome 17. *KRT9* encodes keratin 9 (K9), a type I intermediate filament polypeptide expressed explicitly in terminally differentiated keratinocytes of palmoplantar epidermis [11]. The mutation results in the substitution of leucine (Leu) by serine (Ser) at position 168 in the encoded keratin 9 protein. This missense mutation occurs in a well-defined mutational hotspot of the *KRT9* gene, characterized by high pathogenic variant density with numerous documented reports [12]. *KRT9* has three major domains: The head domain (amino acids 1–153), the central α-helical rod domain (amino acids 154–485) with four helical subsegments (1 A, 1B, 2 A, 2B) and three non-helical linkers (L1, L12, L2), and the tail domain (amino acids 486–623) [13]. This missense mutation occurs in the highly conserved 1 A helical domain of *KRT9*, a functionally critical region for *KRT9* [14]. Pathogenic variants within this 1 A helical domain are predicted to compromise intermediate filament assembly, potentially disrupting the keratinocyte cytoskeleton. This may transform organized filament networks into disorganized meshworks with localized cytoskeletal collapse, ultimately culminating in mechanical fragility of keratinocytes [15]. Clinically, this manifests as palmoplantar epidermal hyperkeratosis, occasionally complicated by congenital interphalangeal joint deformities [16]. Female patients may exhibit elevated risks for ovarian and breast carcinomas [17]. Notably, phenotypic heterogeneity is observed at this identical mutation site, demonstrating variable expressivity across individuals [5]. Histologically, it exhibits epidermolytic hyperkeratosis, marked by perinuclear vacuolization of keratinocytes and large, irregularly shaped keratohyaline granules in the granular layer of the epidermis [16].

Current therapeutic approaches remain limited to symptomatic management. While emollients and systemic retinoids may provide partial relief, their use is constrained by potential adverse effects, including skeletal abnormalities in pediatric patients and teratogenicity in reproductive-aged women. Surgical interventions often prove unsatisfactory due to high recurrence rates and iatrogenic tissue damage [9]. Existing research has validated in vivo CRISPR/Cas9 therapeutics as novel treatment modalities, resulting in significant phenotypic amelioration—including hyperkeratosis (with 50% epidermal thinning), abnormal melanin deposition, and dysregulated stress keratins (K6/K16) while restoring regular differentiation/proliferation markers with minimal off-target effects [18]. We identified a low-frequency pathogenic mutation in the *KRT9* gene within a family, which expands the spectrum of pathogenic mutations associated with *KRT9*-pEDD and lays the groundwork for preimplantation genetic testing for monogenic diseases (PGT-M) for affected individuals in this family.

## Conclusions

In summary, through detailed investigation of a Chinese pedigree with *KRT9*-pEDD, we identified the causative mechanism as a known c.503T > C (p. Leu168Ser) missense mutation in exon 1 of the *KRT9* gene. This variant induced prominent palmoplantar hyperkeratosis in affected family members. Following comprehensive genetic counseling and PGT-M applied to the proband’s embryos, a disease-free embryo was selected for transfer, resulting in the successful delivery of a healthy infant. This study expands the mutational and phenotypic spectrum of *KRT9* variants. It establishes a comprehensive clinical management pathway for prenatal diagnosis and prevention of disease transmission in offspring, providing a reference framework for similar cases.

## Data Availability

No datasets were generated or analysed during the current study.

## Abbreviations

KRT9-pEDD: KRT9-palmoplantar epidermal differentiation disorder
KRT9: Keratin 9
GnRH: Gonadotropin-releasing hormone
ICSI: Intracytoplasmic monosperm microinjection
TE: Trophoblast ectoderm
HGMD: Human gene mutation database
PGT-M: Preimplantation genetic testing for monogenic diseases
CNVs: Copy number variations
QF-PCR: Quantitative fluorescence polymerase chain reaction
DNA: Deoxyribonucleic acid
ACMG: American society for medical genetics and genomics
EXAC: Exome aggregation consortium
